# Prevention of Salivation

**Published:** 1852-11

**Authors:** George Stearns


*Prevention of Salivation.* By George Stearns, M. D. I wish to communicate a fact to you that has recently fallen under my observation, which may be of some interest to the profession generally. All physicians are aware of the salivating effects of calomel, and of the inconvenience that arises from sore mouths and other irritating complaints that affect the patients.

I have had several persons under my care to whom I have been obliged to administer calomel, which I have mixed with supercarbonate of soda, in the proportion of about twice the amount in weight of soda. To one patient in particular, whom I have attended for about ten weeks, I have given three grains of calomel with six grains of soda daily for five weeks, besides administering it frequently during the rest of the time. As yet he has not suffered at all from the salivating effect of the calomel, which has nevertheless been very beneficial to him. It is possible that these were all persons not susceptible to salivation? Or is the absence of salivation to be attributed to the supercarbonate of soda?—*Boston Medical Journal.*

